# Serotonin and Its Receptor as a New Antioxidant Therapeutic Target for Diabetic Kidney Disease

**DOI:** 10.1155/2017/7680576

**Published:** 2017-08-08

**Authors:** Yu Yang, Hui Huang, Zheng Xu, Jun-kai Duan

**Affiliations:** ^1^Department of Endocrinology, Metabolism, and Genetics, Jiangxi Provincial Children's Hospital, Nanchang, Jiangxi, China; ^2^Pediatric Research Institute, Department of Pediatrics, University of Louisville, Louisville, KY, USA; ^3^Department of Cardiovascular Disorders, The First Hospital of Jilin University, Changchun, China; ^4^Department of Cardiovascular Disorders, Jiangxi Provincial Children's Hospital, Nanchang, Jiangxi, China

## Abstract

Diabetic kidney disease (DKD) is a widespread chronic microvascular complication of diabetes mellitus (DM), affects almost 30–50% of patients, and represents a leading cause of death of DM. Serotonin or 5-hydroxytryptamine (5-HT) is a multifunctional bioamine that has crucial roles in many physiological pathways. Recently, emerging evidence from experimental and clinical studies has demonstrated that 5-HT is involved in the pathogenesis of diabetic vascular complications. The 5-HT receptor (5-HTR) antagonists exert renoprotective effects by suppressing oxidative stress, suggesting that 5-HTR can be used as a potential target for treating DKD. In this review, therefore, we summarize the published information available for the involvement of 5-HT and 5-HTR antagonists in the pathogenesis of various diabetic complications with a particular focus of DKD. We conclude that 5-HTR is a potential therapeutic target for treating DKD, as it has been successfully applied in animal models and has currently being investigated in randomized and controlled clinical trials.

## 1. Introduction

Diabetic kidney disease (DKD) is one of the most epidemic chronic microvascular complications of diabetes mellitus (DM), and it is prevalent in approximately 30–50% of patients with diabetes [1–5]. DKD is the leading cause of chronic and end-stage renal diseases worldwide, and in the past few decades, it has been associated with high morbidity and mortality [6–11].

The pathogenesis of DKD remains not completely understood; however, there is strong experimental evidence that prolonged hyperglycemia leads to the mitochondrial production of reactive oxygen species (ROS), resulting in oxidative stress, which plays a key role in DKD [12–16]. Inflammation induced and exacerbated by oxidative stress is closely associated with the development and progression of DKD.

5-Hydroxytryptamine (5-HT) is a potent vasoactive amine that plays pivotal roles in insulin secretion [17–19], energy metabolism [20], mitochondrial biogenesis [21], the immune system [22, 23], and vascular inflammation [24–27]. However, the functions of 5-HT have not been elucidated yet. Recently, several studies have shown that 5-HT and 5-HT receptors (5-HTR) are involved in the pathogenesis of diabetic vascular complications [17, 28–31]. 5-HTR antagonists have a renoprotective effect by suppressing oxidative stress and inflammatory cytokines [32–35], suggesting that 5-HTR antagonists could be used to treat DKD. This review assesses and describes the current understanding of 5-HT's involvement in the pathogenesis of DKD and the potential use of 5-HTR antagonists in the clinical treatment of DKD.

## 2. 5-HT Synthesis and Metabolism and 5-HT Receptors

5-HT is a monoamine neurotransmitter and hormone mainly produced by enterochromaffin cells of the gastrointestinal tract [21]. 5-HT is derived from tryptophan and predominantly stored in circulating platelets, and it is distributed throughout the body to regulate the hormones of several main physiological parameters, such as cardiovascular function [36], insulin secretion [17], energy homeostasis [20], and appetite [37].

**Figure 1:**
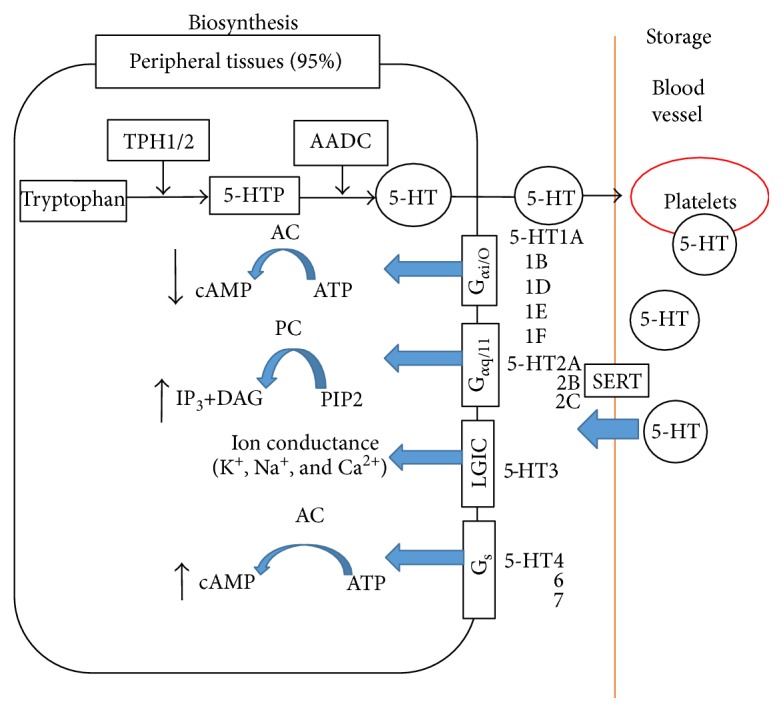
A model of 5-HT biosynthesis and metabolism in peripheral tissues. 5-HT synthesis is dependent on the enzyme tryptophan hydroxylase (TPH); the released 5-HT is controlled by the autonomous nervous system and released locally into the circulatory system, and most of them are stored in platelets. Reuptake of 5-HT is mediated by SERT. The effects of 5-HT are mediated through 14 serotonergic receptors that have been grouped into seven broad families. All 5-HTRs are G protein-coupled receptors (GPCRs), except 5-HT_3_ that is a ligand-gated cationic channel. 5-HT GPCRs were coupled to all three canonical signaling pathways through G_*α*i/O_, G_*α*q/11_, and G_s_ that are involved in the cAMP pathway and allow this receptor family to modulate several biochemical signaling pathways.

5-HT synthesis is dependent on the enzyme tryptophan hydroxylase (TPH), which is encoded by two different genes: tryptophan hydroxylase 1 (Tph1) and Tph2, which are expressed in the peripheral tissues and brain, respectively. Peripheral 5-HT is presumed to be unable to cross the blood-brain barrier. The majority of the peripheral 5-HT is stored in platelets and also present in other tissues and many cells. The released 5-HT is controlled by the autonomous nervous system and released locally into the circulatory system, where it is used for the aggregation of platelets through various stimuli, including atherosclerosis [26, 38]. 5-HT is primarily inactivated by the reuptake of serotonergic neurons that secrete it; this reuptake is mediated by the highly selective plasmalemma 5-HT transporter (5-HTT), which is also known as the serotonin transporter (SERT) [39] (Figure 1).

5-HT produces a myriad of physiological and pathological effects in humans, which are mediated through 14 serotonergic (5-HTergic) receptors that have been grouped into seven broad families (5-HT_1_, 5-HT_2_, 5-HT_3_, 5-HT_4_, 5-HT_5_, 5-HT_6_, and 5-HT_7_). All 5-HTRs are G protein-coupled receptors (GPCRs), except 5-HT_3_ that is a ligand-gated cationic channel. 5-HT GPCR was coupled to all three canonical signaling pathways through G_*α*i_/_O_, G_*α*q/11_, and G_s_ that are involved in the cAMP pathway and allow this receptor family to modulate several biochemical signaling pathways [40].

## 3. 5-HT in Diabetes and Diabetic Complications

Pancreatic *β*-cells synthesize and store 5-HT, which is coreleased with insulin [41]. An increased plasma level of 5-HT is a biomarker for diabetic complications, and positive correlations have been established between the plasma 5-hydroxyindoleacetic acid (5-HIAA; the main 5-HT metabolite) level and coronary heart disease [36, 42–45]. Selective serotonergic functional alterations have shown therapeutic relevance in diabetic rats [29, 30, 46]. These studies and their findings have been summarized in the subsequent sections and suggest that 5-HT plays a role in DM.

### 3.1. 5-HT and Gestational Diabetes

**Figure 2:**
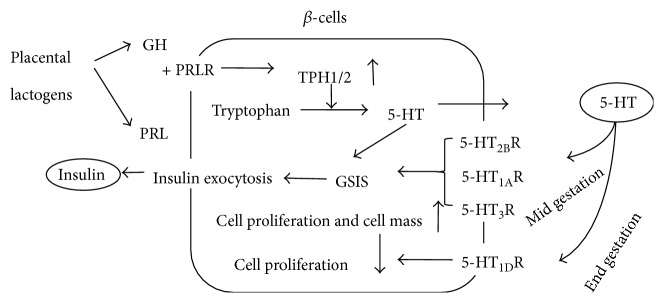
Mechanism of 5-HT in the mouse pancreatic beta-cells during pregnancy. In pregnant mice, prolactin (PRL) stimulates islet prolactin receptors (PRLRs) to trigger a strong upregulation of both isoforms of TPH. TPH upregulation activates 5-HT synthesis in some pancreatic *β*-cells, which in turn induce GSIS. The insulin secretion is upregulated by the 5-HT_2B_ receptor (5-HT_2B_R) and downregulated by the 5-HT_1D_ receptor (5-HT_1D_R) in *β*-cells, making 5-HT a paracrine regulator of *β*-cell proliferation. 5-HT_3A_R channels in wild-type animals allow a 5-HT-mediated influx of cations, depolarizing the resting membrane potential and lowering the threshold for glucose-induced insulin exocytosis.

In pregnant mice, prolactin (PRL) stimulates islet prolactin receptors (PRLRs) to trigger a strong upregulation of both isoforms of TPH. TPH upregulation activates 5-HT synthesis in some pancreatic *β*-cells, which in turn induce glucose-stimulated insulin secretion (GSIS) [47, 48]. The insulin secretion is upregulated by the 5-HT_2B_ receptor (5-HT_2B_R) and downregulated by the 5-HT_1D_ receptor (5-HT_1D_R) in *β*-cells, making 5-HT a paracrine regulator of *β*-cell proliferation. 5-HT_3A_R channels in wild-type animals allow a 5-HT-mediated influx of cations, depolarizing the resting membrane potential and lowering the threshold for glucose-induced insulin exocytosis [19, 49], as illustrated in Figure 2. Disrupting this balance can result in gestational diabetes.

### 3.2. 5-HTR and Type 2 DM

Type 2 DM (T2DM) describes a group of metabolic disorders characterized by defects in insulin secretion and insulin sensitivity. Impaired insulin secretion from pancreatic *β*-cells is an important factor in the etiology of T2DM. However, the complex regulation and mechanism of insulin secretion from *β*-cells have not been completely elucidated.

High plasma levels of 5-HT have been reported in patients with T2DM, although its potential effect on insulin secretion is unclear. The release of 5-HT from activated platelets is enhanced, decreasing intraplatelet 5-HT content and resulting in increased plasma levels of 5-HT in patients with diabetes [44].

#### 3.2.1. 5-HT_2C_R

**Figure 3:**
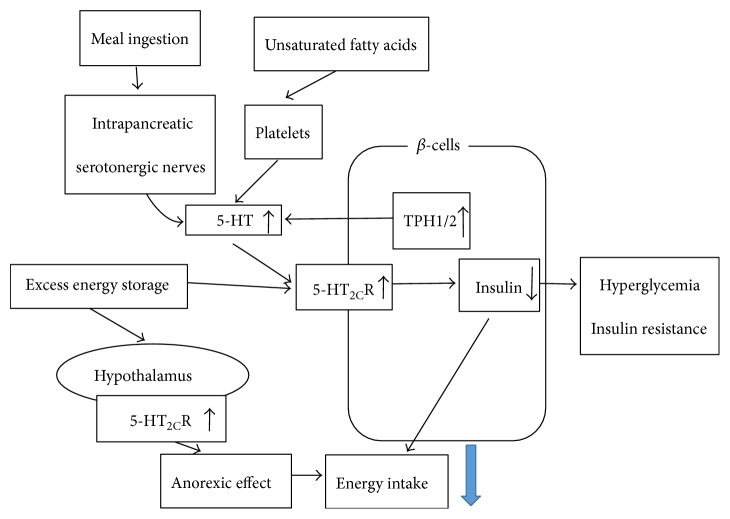
Model showing the modulation of 5-HT2cR in DM. 5-HT2CR-deficient mice showed that 5-HT may affect glucose and lipid metabolism. Insulin secretion is affected by 5-HT2CR, which is indicative of the possibility that an aberrant 5-HT system could also affect the regulation of energy metabolism. Increased expression of 5-HT2CR in both the hypothalamus and *β*-cells could mediate a protective strategy to prevent excess energy intake. 5-HT2CR-expressing pro-opiomelanocortin neurons are required to control energy and glucose homeostasis.

5-HT_2C_R-deficient mice are overweight, exhibit an abnormal feeding behavior, show insulin resistance, and have significantly higher blood glucose concentrations, suggesting that 5-HT may affect glucose and lipid metabolism [17, 20, 50]. Insulin secretion is affected by 5-HT_2C_R, which is indicative of the possibility that an aberrant 5-HT system could also affect the regulation of energy metabolism. Increased expression of 5-HT_2C_R in both the hypothalamus and *β*-cells could mediate a protective strategy to prevent excess energy intake. As illustrated in Figure 3, 5-HT_2C_R-expressing pro-opiomelanocortin neurons are required to control energy and glucose homeostasis [51].

Although, in human T2DM islet cells, the expression of 5-HT_2C_R has not been observed [31], the 5-HT_2C_R agonist Belviq (lorcaserin) is the first FDA-approved drug to treat obesity in 15 years [52], and central serotonin 2C receptors regulated glucose homeostasis and may represent a rational target for type 2 diabetes (T2DM) treatment [53, 54]. The 5-HT_2C_R agonist m-chlorophenylpiperazine (mCPP) improves glucose homeostasis and insulin sensitivity, and antagonists or genetic loss of 5-HT_2C_R impairs glucose homeostasis [55, 56].

#### 3.2.2. 5-HT_1D_R and 5-HT_1A_R

**Figure 4:**
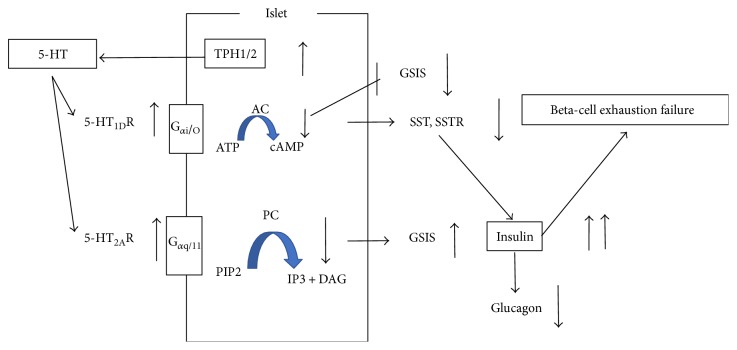
Illustration to show the mechanism of 5-HT1D and 5-HT2AR in human T2DM. 5-HT1DR and 5-HT1AR messenger RNA expression was increased in human T2DM islets. The 5-HT2AR antagonist (sarpogrelate hydrochloride) markedly decreased the glycated hemoglobin A1c level. The expression of 5-HT1DR had a negative correlation with somatostatin (SST) and SST receptors (SSTR), whereas the expression of 5-HT2AR did not have any correlation with either SST or any of the SSTRs; this suggests that increased expression of HT1DR in human islet cells, as observed in T2DM islet cells, leads to decreased expression of SST and its receptors.

Bennet et al. [31] reported that 5-HT_1D_R and 5-HT_1A_R messenger RNA expression was increased in human T2DM islets. 5-HT inhibits both basal- and glucose-induced insulin secretions, and the selective 5-HT_1D_R agonist (PNU142633) inhibits GSIS in nondiabetic human islets, whereas the 5-HT_1D_R antagonist (LY310762) stimulates GSIS. Interestingly, upon stimulation with 5-HT in isolated islets from patients with T2DM, the inhibitory effect of 5-HT was completely lost (both in basal and stimulatory conditions of glucose); instead, the stimulation of insulin secretion was observed. This indicated that 5-HT acts through increased signaling through the 5-HT_2A_R in diabetic conditions. The 5-HT_2A_R antagonist (sarpogrelate hydrochloride) markedly decreased the glycated hemoglobin A1c level. The expression of 5-HT_1D_R had a negative correlation with somatostatin (SST) and SST receptors (SSTR) 1–5, whereas the expression of 5-HT_2A_R did not have any correlation with either SST or any of the SSTRs; this suggests that increased expression of HT_1D_R in human islet cells, as observed in T2DM islet cells, leads to decreased expression of SST and its receptors (Figure 4).

### 3.3. 5-HT as an Immunomodulator in DM

Although several physiological causes that lead to DM remain unknown, evidence suggests that autoimmunity plays an important role in DM and diabetic complications. There is an increasingly collective perspective regarding the association of 5-HT with the activation of immunoinflammatory pathways and the onset of autoimmune reactions. Almost all the circulating 5-HT are found in platelets and released following platelet activation, on contact with damaged endothelium or induced by ischemia, indicating that 5-HT also contributes to the innate and adaptive immune responses [22, 57]. 5-HT stimulation increases murine peritoneal macrophage production of proinflammatory cytokines [25]. The expression of 5-HTRs has been identified in rodent and human innate immune cells, which include neutrophils, eosinophils, monocytes, macrophages, dendritic cells, mast cells, and natural killer cells [58].

5-HT was identified as an immunomodulator owing to its ability to stimulate or inhibit inflammation. Moreover, 5-HT has immunomodulatory effects that are induced by activating 5-HTR and SERT, which are differentially expressed in many leukocytes. Arthritis [59], systemic sclerosis [38, 60], lung fibrosis [61], and allergic asthma [62] are all associated with changes in the serotonergic system, which is associated with leukocytes.

### 3.4. 5-HT_2A_R and DM-Induced Vascular Complications

5-HT is a potent vasoactive amine in the cardiovascular system. Cardiovascular disorders of diabetes can be characterized by atherosclerosis [63]. There is strong evidence that impaired vascular endothelial and smooth muscle functions play important roles in the process of DM-induced cardiovascular complications [63, 64]. 5-HT, induced by impaired vascular endothelial cells, is involved in the pathological process of platelet aggregation [45], thrombogenesis [65], contraction of carotid arteries [66], and arteriogenesis [67] in DM-induced vascular complications through 5-HT_2A_R.

**Figure 5:**
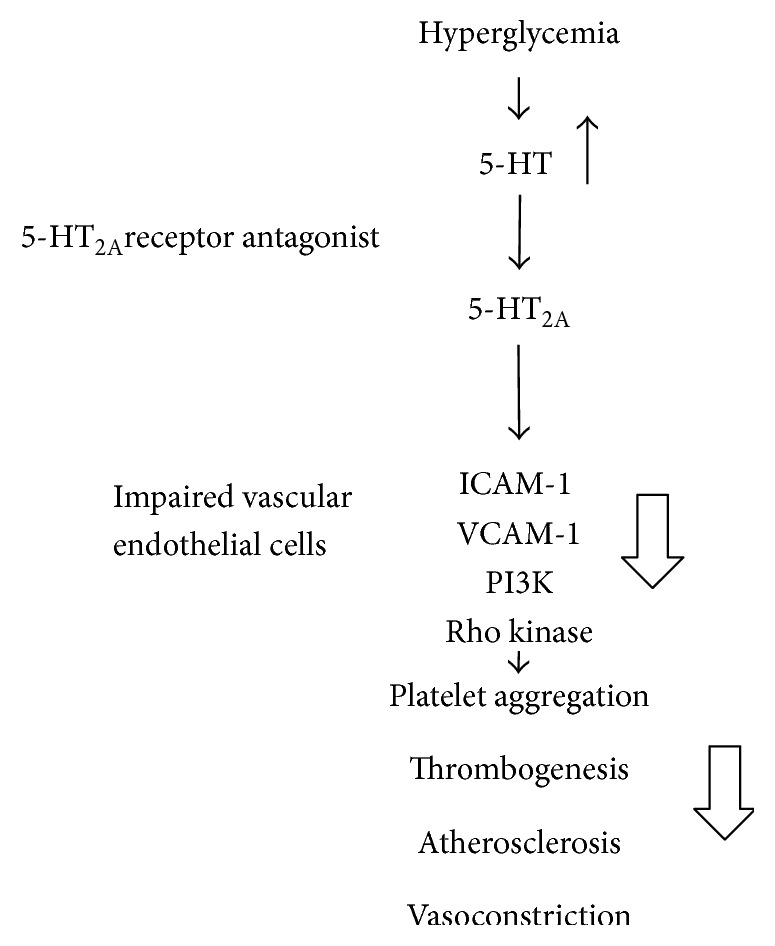
Mechanisms of 5-HT_2A_ receptor antagonist contributing to DM-induced cardiovascular complications. Sarpogrelate, a 5-HT2AR antagonist, has been shown to attenuate diabetes-induced cardiovascular complications, which decrease the blood glucose level, inhibit the release of intercellular adhesion molecule-1 (ICAM-1) and vascular cell adhesion molecule-1 (VCAM-1), and reduce 5-HT-induced contraction in aortas through the PI3K and Rho kinase pathway.

Sarpogrelate, a 5-HT2AR antagonist, has been shown to attenuate diabetes-induced cardiovascular complications, which decrease the blood glucose level [66], inhibit the release of intercellular adhesion molecule-1(ICAM-1) and vascular cell adhesion molecule-1(VCAM-1) [68], and reduce 5-HT-induced contraction in aortas through the PI3K [66] and Rho kinase [69] pathway, as illustrated in Figure 5.

## 4. Mechanism of the 5-HTR Antagonist for Treating DKD

DKD is a main microvascular complication of diabetes and the most common cause of end-stage renal disease strongly associated with cardiovascular morbidity and mortality, which cause an enormous burden on affected patients and health care systems [70]. Histopathological changes associated with DKD are characterized by thickening of the glomerular basement membrane; podocyte effacement and hypertrophy; accumulation of extracellular matrix and proteins, such as collagen and fibronectin; and the hyalinization of afferent and efferent glomerular arterioles [71, 72].

Studies have indicated that inflammation is an important mechanism in the pathogenesis of DKD that triggers a complex network of pathophysiological events that modulate intracellular signaling pathways involving protein kinase C [73–75] and ROS [76–78] and act in a concerted manner to induce transcription factors, cytokines, chemokines, and growth factors during hyperglycemia [13–15, 79–81]. Although many factors have been implicated in the pathogenesis of DKD, inflammation is believed to play a fundamental role in the early development and progression of DKD [14, 71, 78, 80, 82]. Drugs with anti-inflammatory effects have been used as a new clinical approach for treating DKD.

**Figure 6:**
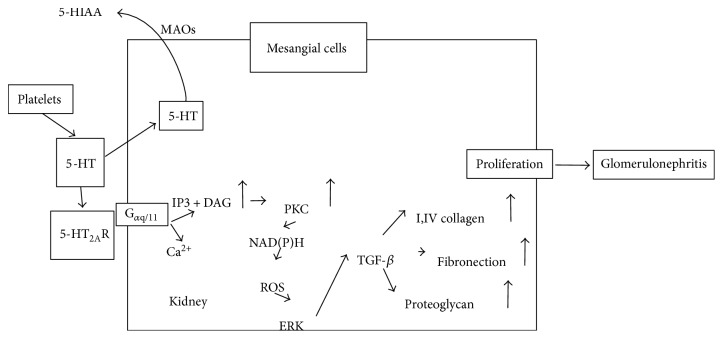
Illustration to show the mechanism of 5-HT_2A_R in mesangial cells. 5-HT has been shown to enhance the production of type IV collagen by human mesangial cells, and its production is mediated by the activation of protein kinase C and a subsequent increase in active TGF-*β*. Stimulation of 5-HT2ARs by 5-HT induces the expression of TGF-*β* through extracellular signal-regulated kinases.

Previous reports have indicated that the increased plasma concentrations of 5-HT or its metabolite (5-HIAA) are valuable biomarkers for estimating the DKD-associated risk during the early stages of the disease [35, 83, 84]. 5-HT has been shown to enhance the production of type IV collagen by human mesangial cells, and its production is mediated by the activation of protein kinase C and a subsequent increase in active transforming growth factor-*β* (TGF-*β*) [85]. Stimulation of 5-HT_2A_Rs by 5-HT induces the expression of TGF-*β* through extracellular signal-regulated kinases, a key mediator of proliferative and fibrotic signals in mesangial cells [86–89], as illustrated in Figure 6.

Studies have shown that 5-HTR antagonists are effective in preventing diabetic nephropathy. Sarpogrelate, a 5-HT2 subtype 2A antagonist [33, 34], reduced albuminuria in the early stages of DKD by improving glomerular endothelial function through the reduction in glomerular platelet activation and an increase in serum adiponectin concentrations in a diabetic animal model. Ogawa et al. [90] and Park et al. [91] found that sarpogrelate can reduce albuminuria and plasma and urinary monocyte chemoattractant protein-1 levels in patients with DKD. Tropisetron, a 5-HT_3_ receptor antagonist, can attenuate early DM through calcineurin inhibition and by suppressing oxidative stress and some inflammatory cytokines in streptozotocin-induced diabetic rats [32].

## 5. Conclusions

There is an increasing repertoire of evidence supporting 5-HT as a causative agent for increased ROS generation in DM. Since 5-HT mediates accelerated atherosclerosis in diabetes, pharmacological inhibition of the 5-HT receptor presents an attractive therapeutic strategy for patients with diabetes to attenuate the development of nephropathy and macrovascular complications. A better understanding of the role of these new receptor targets in the context of DKD will facilitate the development of novel therapeutic strategies that can be successfully translated into clinical applications.

## Abbreviations

5-HT: 5-Hydroxytryptamine
TPH: Tryptophan hydroxylase
AADC: Unbiquitous aromatic L-amino acid decarboxylase
5-HTT: 5-HT transporter
Cys-loop LGICs: Cys-loop ligand-gated ion channels
5-HTT (SERT): 5-Hydroxytryptamine transporter
GPCRs: G protein-coupled receptors
AC: Adenylyl cyclase
cAMP: Cyclic adenosine monophosphate
PIP2: Phosphatidylinositol 4,5-bisphosphate
IP3: Inositol trisphosphate
DAG: Diacylglycerol
PRL: Prolactin
PRLR: Islet prolactin receptors
GSIS: Glucose-stimulated insulin secretion
SST: Somatostatin
SSTR: SST receptors
Rho: Ras homolog gene family member
ICAM-1: Intercellular adhesion molecule-1
VCAM-1: Vascular cell adhesion molecule-1
TGF-*β*: Transforming growth factor-*β*
PKC: Protein kinase C
ROS: Reactive oxygen species
NADP: Nicotinamide adenine dinucleotide phosphate
ERK: Extracellular signal-regulated kinases.
